# Supplementary material for: The Impact of Urban Allotment Gardens on Physical and Mental Health in Norway
Source: Int J Environ Res Public Health. 2024 May 31;21(6):720. doi: 10.3390/ijerph21060720 (PMC11204083; doi:10.3390/ijerph21060720)
Supplement: Supplementary file 1 [file ijerph-21-00720-s001.zip › ijerph-3017530-supplementary.pdf]

Interview Guide: Allotment Gardens

Mapping of General Information

Gender:
Education Level:
Age:
18-30
31-55
56-90

Attachment to and living in Allotment Gardens

1. When did you acquire your allotment garden?
2. Did you wait on a waiting list before being assigned a parcel? If so, how long did it take before you received your parcel?
3. Do you spend the entire season in the allotment garden, or are you mostly there on weekends or holidays?
4. During a season, how much time do you typically spend in your allotment garden? Who do you share this time with (visitors, partner, family, etc.)?

Lifestyle in the allotment garden

- What does a typical day in the allotment garden look like for you? What are you doing?
- Sometimes various social events are held in the allotment garden, do you tend to attend them? If so, what events do you choose? (Dugnad, apple pressing, social arrangements, film viewing ect.)
- How is your lifestyle affected by owning an alloment garden? Is there any specific interests that you are doing in the garden?
- Are there any disadvantages when your living in the allotment garden?
- Do you feel that it is easy to be part of the community in the allotment garden? And do you think it is easy for new people to get in touch with the environment in the garden?
- What liability is to own an allotment garden? What does this mean for you?
- Have you gained any new knowledge/ experience after you got a allotment garden? If so, what is it? (Plants, cultivation, maintenance ect.)
- Is there any kind of noise that has bothered you while you have owned an allotment garden? (traffic, neighbors, animals ect.)

Lifestyle in the Allotment Garden

1. Describe a typical day in your allotment garden. What activities do you engage in?
2. Do you participate in various social events held in the allotment garden (e.g., communal work, apple pressing, social gatherings, film viewings)? If so, which events do you attend?
3. How has owning an allotment garden affected your lifestyle? Are there specific interests or hobbies you pursue in the garden?
4. Are there any disadvantages associated with living in the allotment garden?
5. Do you find it easy to become part of the community in the allotment garden? Is it accessible for new people to connect with the environment there?
6. What responsibilities come with owning an allotment garden, and how do these impact you personally?

Gained Knowledge and Experiences

1. Have you acquired new knowledge or experiences related to plants, cultivation, or maintenance since obtaining your allotment garden? If so, what have you learned?
2. Have any specific noises (e.g., traffic, neighbors, animals) bothered you while owning an allotment garden?

Experienced Meaning of Allotment Gardens

1. When and how did you first learn about allotment gardens in Oslo?
2. Has your perception of allotment gardens changed since becoming an owner? If so, how?
3. Do you believe that Oslo (and other large cities) needs more allotment gardens? Why or why not?
4. Would your interest in having an allotment garden be the same without a cabin (e.g., just a garden parcel)?
5. Compare your home with the allotment garden you own. Do you have a garden at home, or do you live in an apartment?
6. What impact would be losing the opportunity to own an allotment garden have on you (both physically and mentally)?

Conclusion

1. Is there anything else you'd like to share that we haven't discussed during this interview?
